# Ejaculatory function after robotic waterjet ablation for the treatment of benign prostatic hyperplasia: a systematic review

**DOI:** 10.1038/s41443-025-01087-6

**Published:** 2025-05-14

**Authors:** Anthony Bettencourt, Jordan Wu, Joseph A. Borrell, Thiago P. Furtado, Jesse N. Mills, Rajiv Jayadevan, Sriram V. Eleswarapu

**Affiliations:** 1 David Geffen School of Medicine, University of California, Los Angeles, CA USA; 2 Division of Andrology, Department of Urology, David Geffen School of Medicine at UCLA, Los Angeles, CA USA

**Keywords:** Surgery, Sexual dysfunction

## Abstract

Robotic waterjet ablation (RWJA), known by the trade name of Aquablation, is a minimally invasive, heat-free technique for treating benign prostatic hyperplasia (BPH) that offers comparable efficacy to transurethral resection of the prostate (TURP). Unlike TURP, RWJA utilizes targeted tissue mapping, potentially enhancing the preservation of sexual function, particularly antegrade ejaculation. This systematic review evaluated sexual outcomes following RWJA, emphasizing ejaculatory dysfunction and antegrade ejaculation preservation. A literature search conducted through January 1, 2025, in PubMed, Embase, and Cochrane databases identified 15 studies involving 1533 patients. Preservation rates of antegrade ejaculation post-RWJA ranged from 72 to 99.6%. Erectile function remained stable across all reviewed studies. Notably, a randomized controlled trial comparing RWJA to TURP demonstrated significantly lower rates of ejaculatory dysfunction in the RWJA group, maintained for up to five years. Despite promising findings indicating durable preservation of ejaculatory function, there remain limitations due to a scarcity of randomized controlled trials and limited long-term follow-up beyond 12 months. Future comparative studies evaluating RWJA against other minimally invasive BPH treatments are needed to further validate these findings and better define the sexual function outcomes associated with this innovative procedure.

## Introduction

Benign prostatic hyperplasia (BPH) is the most common benign neoplasm affecting men in the United States [1]. BPH can lead to bothersome lower urinary tract symptoms (LUTS), which become more prevalent as men age [2]. The historical gold standard surgical treatment for LUTS related to BPH is transurethral resection of the prostate (TURP) [3]. Although TURP is effective at alleviating LUTS, it is associated with undesirable sexual side effects, particularly retrograde ejaculation (RE), which occurs in up to 66.1% of patients [4]. RE can be particularly concerning to men who are contemplating surgical intervention for LUTS. Bouhadana et al. reported that 92% of men undergoing surgical treatment for BPH considered maintenance of ejaculatory function to be important, especially those aged 50–59 [5]. Although men over 60 were more concerned with urinary incontinence overall, over 60% still reported worry regarding the treatment’s impact on their ejaculatory function. Therefore, the preservation of ejaculatory function remains an important consideration in surgical management decisions for men with BPH.

In recent years, several novel, minimally invasive BPH treatments have emerged to help preserve sexual function by limiting collateral damage to surrounding structures during BPH surgery. Robotic waterjet ablation (RWJA), known by the tradename Aquablation, (PROCEPT BioRobotics, Redwood Shores, CA, USA), was first described in 2016 [6] and approved by the United States Food & Drug Administration in 2017. It has demonstrated comparable efficacy to TURP in improving LUTS and is now included in the American Urological Association (AUA) guidelines as a recommended therapy [7]. Unlike TURP, RWJA uses a heat-free approach with high-pressure waterjet technology, which, in combination with transrectal ultrasound, allows for the precise mapping of prostatic tissue. This approach reduces damage to surrounding structures, including the seminal colliculus, which, when affected, can contribute to ejaculatory dysfunction (EjD). Unlike other therapies that incidentally preserve sexual function, RWJA is the first surgical treatment intentionally designed to preserve ejaculation through target surgical planning and tissue mapping [6]. This differentiates RWJA from other BPH treatment options, including office-based minimally invasive surgical therapies (MIST) such as prostatic urethral lift (PUL) and water vapor thermal therapy (WVTT) which are also associated with low ejaculatory risk [8, 9]. The deliberate design features of RWJA set it apart from both standard surgical interventions such as TURP and MIST approaches.

A limited number of recent systematic reviews have examined sexual health outcomes after RWJA [10, 11]. Our review adds to the existing literature, incorporating additional studies with longer-term follow-up, including the 5-year evaluation from the pivotal WATER randomized controlled trial (RCT) [12]. This review aims to assess sexual health outcomes associated with RWJA, with a particular focus on EjD and the preservation of antegrade ejaculation.

## Methods

### Search strategy

To address our primary outcome of interest, EjD, we performed a comprehensive literature search across PubMed, Embase, and the Cochrane databases, including all studies published up to January 2025. The following search terms were used and pooled together with the Boolean operator “OR”: “Aquablation”, “Aquabeam”, and “waterjet ablation” to identify studies involving RWJA; “ejaculatory function”, “ejaculatory dysfunction”, “sexual function”, “male sexual health”, “retrograde ejaculation”, “antegrade ejaculation”, “MSHQ”, “SHIM”, and “IIEF” to identify studies reporting on sexual outcomes. Each of these subgroups was then pooled together with the Boolean operator “AND”. We included English-language studies on RWJA that reported the rate or incidence of EjD. Case reports, case series, abstracts, reviews, and editorials were excluded. Secondary analyses of already included studies were excluded unless they provided additional information on ejaculatory function. Due to the limited number of RCTs and prospective studies on sexual function after RWJA, retrospective studies were included for a broader review.

### PRISMA selection and detailed analyses

Articles identified through the search strategy were uploaded to Rayyan [13], a web-based tool for systematic reviews, for selection based on PRISMA methodology [14]. Rayyan utilizes artificial intelligence to assist with article screening. For our purposes, this feature was used to help identify duplicates during the selection process. Titles and abstracts were reviewed for initial inclusion, and preliminary exclusions were made based on this review. Relevant articles were retrieved in full-text form for a more detailed analysis, and further exclusions were made. Two authors (AB, JW) independently screened all abstracts. Consensus was achieved by a third author (RJ). Data extraction was performed using a standardized form and captured study design, study period, cohort size, follow-up duration, ejaculatory function measures, and sexual adverse effects. For studies that reported the rate of EjD, this was converted to the rate of antegrade ejaculation preservation for consistency. Due to the anticipated heterogeneity of outcome measures (e.g., Male Sexual Health Questionnaire (MSHQ) [15], Male Sexual Health Questionnaire Ejaculatory Dysfunction Short Form (MSHQ-EjD) [16], International Index of Erectile Dysfunction Long Form (IIEF-15) [17], Clavien-Dindo classification [18]), a meta-analysis was not performed, and results were instead synthesized narratively.

## Results

### Study selection

The bibliographic search yielded 342 records: 79 from PubMed, 212 from EMBASE, and 51 from Cochrane. After the removal of 153 duplicate records, 189 unique records were screened for eligibility. During the initial screening process, 161 records were excluded for the following reasons: 93 were non-clinical studies, 60 were abstracts, 4 were case series or case studies, 3 were additional duplicates, and 1 was a non-English language study.

Subsequently, 28 full-text articles were sought for retrieval and assessed for eligibility. Seven were excluded as secondary analyses of already included data, offering no relevant additional information. Of these, four were subsequent analyses of specific country-level data focusing exclusively on the US, Canadian, or UK cohorts [19–22]. As we were interested in evaluating the primary data for entire sample sizes, we chose not to include these secondary or subsidiary analyses. One was a case-control study examining video logs from the WATER and WATER II clinical trials to determine which anatomic structures should be preserved for antegrade ejaculation [23]. While novel, this did not provide additional relevant information on EjD, besides observations on technique. Another was a subgroup analysis of the WATER trial examining prostates 50–80 mL, with sexual health data already reported in the original study [24]. The final excluded study was a comparative analysis of the WATER and WATER II trials, but the additional sexual health data were already present in the original publications [25].

Ultimately, we retrieved data for men in 15 different studies with results in 21 separate published articles that met the inclusion criteria. For the WATER trial, there were four follow-up studies at 6, 24, 36, and 60 months [12, 26–28]. These represent longitudinal data from the same patient cohort and were not treated as independent populations. Accordingly, only the total number of unique patients from the original WATER cohort was included in the final patient count.

**Fig. 1:**
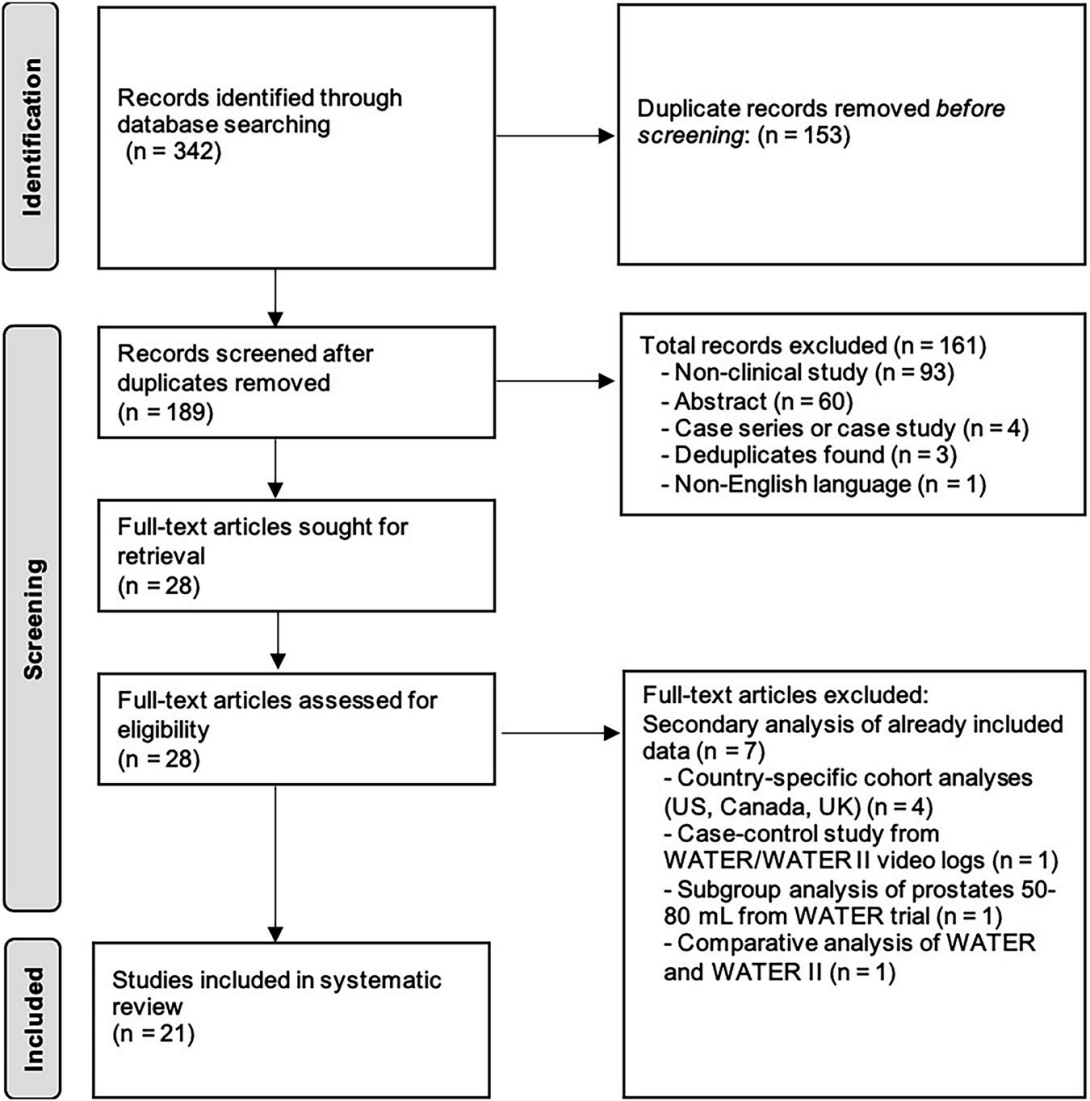
Detailed PRISMA flow diagram of article selection.

Additionally, two of the included studies were pooled analyses of the WATER and WATER II cohorts, and one was a subgroup analysis of the WATER cohort [29, 30]. These studies were deemed relevant for inclusion as the additional information they provided was not included among the original clinical trial data, and they provided further useful insights. These patients were not included in the final calculation of the total number of patients included in the review. The detailed study selection flow is outlined in Fig. 1.

### Study characteristics and outcome measures

Each of the 15 included studies is summarized in Table 1. The total number of patients included was 1533. Most (14/15) of the studies reported the study period, with follow-up durations ranging from 3 to 60 months (median 12, IQR 6). Most studies had cohorts of over 100 participants, with only two having 20 or fewer. There was only one RCT, the WATER trial, which compared RWJA to TURP. However, there were three additional registered clinical trials: WATER II, FRANCAIS WATER, and OPEN WATER [31–33]. Two of the studies were retrospective observational studies [34, 35]. The remaining were prospective observational designs, one of which compared RWJA to holmium laser enucleation of the prostate (HoLEP) [36].

**Table 1:** Sexual health outcomes after RWJA.

Study (NCT; Trial Name)	Study design	Study period	Cohort size	Follow-up time	Prostate size	Sexual function outcomes and adverse events	Antegrade ejaculation preservation
Gilling et al. [6]	Prospective, non-randomized, single-center clinical trial	1/2013–2/2014	15	6 months	Not specified	No reported incidence of retrograde ejaculation or erectile dysfunction.	Not reported
Gilling et al. [40]	Prospective, non-randomized, multi-center clinical trial	11/2013–7/2014	21	12 months	25–80 ml	On IIEF-15, no statistically significant change in any score except for an increase in intercourse satisfaction (*p* < 0.01). No decrease in questions 9 and 10 of the IIEF-15, which assess EjD.	Not reported
Gilling et al. [26], Gilling et al. [27], Gilling et al. [28], Gilling et al. [12] (NCT02505919; WATER I)	Double-blind, multi-center, randomized controlled trial	10/2015–12/2021	181: 116 RWJA, 65 TURP	6 months	30–80 g	6-month results: Threshold decreases in MSHQ-EjD or IIEF-5 scores in 33% of RWJA and 56% of TURP cases (*p* = 0.0268). On IIEF-15, overall sexual satisfaction for RWJA was significantly better (*p* = 0.0492). EjD scores on MSHQ-EjD stable after RWJA but significantly worse after TURP (*p* = 0.0254).	RWJA vs TURP at 6 months: 90 vs 64%, *p* = 0.0003. When post treatment cautery avoided: 93 vs 84%, *p* = 0.2616. For prostates over 50 ml: 98 vs 59%, *p* = 0.0001.
						24-month results: EjD scores on MSHQ-EjD significantly worse after TURP compared to RWJA at 1 year (*p* = 0.0116) and 2 years (*p* = 0.0474). No de novo erectile dysfunction events in either arm.	Not reported
						36-month results: MSHQ-EjD averaged 2.8 points lower at all fu visits for TURP when compared to RWJA (*p* = 0.0008). No significant change in MSHQ-EjD for Aqua. IIEF-15 showed no significant differences in either group or between the two groups.	Not reported
						60-month results: At 5 years, MSHQ-EjD averaged 2.7 points lower at all fu visits for TURP when compared to RWJA (*p* = 0.0015). No significant change in IIEF-5 for either group.	Not reported
Misrai et al. [32] (NCT03191734; FRANCAIS WATER)	Prospective, non-randomized, multi-center clinical trial	9/2017–12/2017	30	12 months	30–80 ml	No significant change on MSHQ-EjD, MSHQ bother or IIEF-15 between baseline and follow up visits. No patient reported any incidence of de novo erectile dysfunction.	73.3% at 12 months
Bhojani et al. [31] 2019(NCT03123250; WATER II)	Prospective, non-randomized, multi-center clinical trial	9/2017–12/2017	101	12 months	80–150 ml	MSHQ-EjD 8.1 (95% CI: 7.2–9.0) at baseline to 7 (5.8–8.3) at 3 mo and 6.6 (5.4–7.9) at 12 months. 3 and 12 month decreases met study’s noninferiority hypothesis (*p* = .0026). IIEF-5 scores were unchanged from baseline (15.1) to 12-months (16.3). IIEF-15 showed no major changes in any category. No patient reported any de novo erectile dysfunction.	81% at 3 months
Bach et al. [37]	Prospective observational study	9/2017–6/2018	118	3 months	Not specified	No survey data reported, though antegrade ejaculatory preservation was assessed with MSHQ-EjD.	73% at 3 months
Bach et al. [33] (NCT02974751; OPEN WATER)	Prospective, non-randomized, multi-center, open-label clinical trial	9/2017–12/2018	178	12 months	20–150 ml	MSHQ-EjD 8 at baseline to −1 at 3 months (*p* = 0.0994) and −1.1 at 12 months (*p* = 0.0884). No significant change in IIEF-15. One reported case of ED with 1 point drop on IIEF-5. MSHQ bother changed by −0.3 and −0.7 points at 3 and 12 months (p = 0.0962 and 0.0025).	92% at 12 months
Labban et al. [38]	Prospective observational study	3/2018–3/2020	59	12 months	Not specified	No survey data reported, though antegrade ejaculatory preservation was assessed with MSHQ-EjD.	82.8% at 3 months; 84.6% at 12 months
D’agostino et al. [34]	Retrospective observational study	6/2019–7/2020	53	3 months	Not specified	No validated surveys used. Antegrade ejaculatory preservation was assessed through patient self-report.	72% at 3 months
Yee et al. [42]	Prospective observational study	6/2019–9/2020	20	6 months	Not specified	IIEF-5 16.1 at baseline to 14.9 at 3 months (*p* = 0.953) to 16.6 at 6 months.	75% at 3 months
El Hajj et al. [35]	Retrospective observational study	9/2017–8/2020	175	12 months	Not specified	No validated surveys used. Antegrade ejaculatory preservation was assessed through patient self-report.	81.4% at 12 months
Omidele et al. [41]	Prospective observational study	12/2019–12/2023	330	48 months	38–293 ml	No validated surveys used. Antegrade ejaculatory preservation was assessed through patient self-report.	99.6% at 48 months
Michaelis et al. [36]	Prospective observational study	6/2020–4/2022	40: 16 RWJA, 24 HoLEP	12 months	Not specified	MSHQ-EjD 9.86 at baseline for RWJA and 9.41 for HoLEP. At 3 mos, 8.57 vs 4.42 (p = 0.02). At 6 months 7.50 versus 4.56; At 12 months 7.33 versus 4.41.	Not reported
Hinata et al. [43]	Prospective observational study	n/a	103	6 months	Not specified	No reported incidence of erectile dysfunction or EjD.	Not reported
Amparore et al. [39]	Prospective observational study	10/2018–4/2023	109	12 months	<80 ml	MSHQ-EjD 18 at baseline to 17 at 1 month (*p* = 0.45) to 17 at 3 months (*p* = 0.87) to 16 at 6 months (*p* = 0.45) to 17 at 12 months (*p* = 0.45). IIEF-5 24 at baseline to 25 at 1 month (*p* = 0.98) to 25 at 3 months (*p* = 0.98) to 25 at 6 months (*p* = 0.98) to 24 at 12 months (*p* = 0.98).	97% at 12 months

*RWJA* robotic waterjet ablation, *EjD* ejaculatory dysfunction, *IIEF-15* international index of erectile function long form, *IIEF-5* international index of erectile function short form, *MSHQ-EjD* male sexual health questionnaire ejaculatory dysfunction short form, *TURP* transurethral resection of the prostate, *fu* follow-up, *HoLEP* holmium laser enucleation of the prostate.

Our primary outcome measure of interest was EjD. However, there was variation among the studies in how this was described and evaluated. Most studies utilized the MSHQ-EjD or MSHQ (8/15) [12, 26–28, 31–33, 36, 37–39]. One used questions 9 and 10 on the IIEF-15 (1/15) [40]. Six studies reported EjD solely as an adverse event [6, 34, 35, 41–43]. Only two studies reported MSHQ bother results (2/15) [32, 33].

### Patient inclusion and exclusion criteria within published studies

Patient inclusion criteria varied across the studies. Most commonly, enrolled participants were 45–80 years of age, had an International Prostate Symptom Score (IPSS) [44] ≥12, and a peak urinary flow rate (Qmax) of ≤12–15 mL/s. Prostate size enrollment criteria differed, though most studies evaluated patients with prostate volumes of 25–80 mL as measured by transrectal ultrasound (TRUS). One study specifically evaluated larger prostates measuring 80–150 mL [31]. Two evaluated a broad range of prostate sizes, including 20–150 mL and 38–293 mL [33, 41]. Other studies had no volume requirements, instead enrolling any patient who was planned to or had already undergone an RWJA procedure. Patients were generally required to have failed prior medical therapy or to have moderate-to-severe BPH symptoms, with most studies defining this as IPSS ≥ 12. Another study enrolled patients who had acute urinary retention (AUR) and had persistent catheter-dependence [42]. Exclusion criteria were similar across studies and excluded those patients with significant comorbidities, such as prostate or bladder cancer or other uncontrolled chronic diseases.

### Sexual function outcomes: validated questionnaires

The earliest available data on RWJA’s impact on sexual function with validated questionnaires comes from Gilling et al. in [40]. Among 21 participants, this study found no significant change in any IIEF-15 score, except for an increase in intercourse satisfaction (p < 0.01) [40]. In this study, there were no decrements on IIEF-15 questions 9 and 10, which assess EjD, and no cases of RE.

The WATER I trial, a prospective double-blind multicenter RCT, provided the first large-scale comparison between RWJA and TURP. At 6 months, MSHQ-EjD or IIEF-5 scores declined in 33% of RWJA patients versus 56% of TURP patients (p < 0.05) [26]. Overall sexual satisfaction scores were also significantly better after RWJA (p < 0.05), and MSHQ-EjD scores remained stable after RWJA but worsened after TURP (p < 0.05). MSHQ-EjD scores remained significantly worse in the TURP arm compared to RWJA at 12 months (p < 0.05), 24 months (p < 0.05), and 36 months (p < 0.01) [27, 28]. At 5 years, the scores were significantly worse in the TURP cohort across all follow-up visits (p < 0.01) [12]. Those who underwent RWJA reported minimal changes in ejaculatory function, as measured by MSHQ-EjD, at all time points out to 5 years.

In a subgroup analysis of the WATER I trials for prostates 50–80 mL, there were no significant changes seen in MSHQ-EjD in the RWJA group, while the TURP group saw an average decline across all study visits up to 5 years (0.6 ± 4.8 vs. −2.1 ± 5.0, p < 0.01) [29].

Following the WATER trial, the WATER II trial was conducted to investigate RWJA in men with larger prostates (80–150 mL). The results demonstrated a slight decline in mean MSHQ-EjD from 8.1 at baseline to 7.0 at 3 months and 6.6 at 12 months [31]. However, these changes met the study’s noninferiority hypothesis (p < 0.01) and were deemed non-significant. Mean IIEF-5 scores remained stable from baseline (15.1) to 12 months (16.3), and no patient reported new-onset erectile dysfunction.

Recently, additional analyses of various subgroups have been published that compare the findings between WATER and WATER II. One study compared younger men (<65 years) to elderly men (>65 years). At baseline, younger men demonstrated higher scores on the MSHQ-EjD when compared to elderly men, though this difference was not statistically significant (8.7 ± 3.7 vs. 7.7 ± 3.8) [30]. At subsequent follow-up visits up to 36 months, there was no statistically significant change in MSHQ-EjD at any visit when compared across the two groups. In contrast, IIEF-5 scores showed significant improvements over time, particularly in younger men. At baseline, there was no statistically significant difference in IIEF-5 scores between younger men and elderly men (17.3 ± 7.2 vs. 15.4 ± 6.8). By the three-year follow-up, both younger men (20.4 ± 4.9) and elderly men (16.4 ± 6.2) saw their scores increase, with the change seen in younger men being significantly improved compared to elderly men (p < 0.05). A further study evaluated the effect of single versus multiple passes on RWJA outcomes from both WATER and WATER II and found no significant difference in the risk of EjD based on the number of passes [45].

Additional studies have continued to demonstrate the preservation of sexual function with RWJA. FRANCAIS WATER, a clinical trial conducted in France, included 30 patients with a 12-month follow-up and had similar inclusion criteria to WATER I. They demonstrated no significant changes in MSHQ, MSHQ bother, or IIEF-15 scores between baseline and follow-up visits [32]. OPEN WATER, a multicenter open-label clinical trial evaluating prostates from 20–150 mL, demonstrated that MSHQ-EjD scores declined slightly at 3 and 12 months, though this was not statistically significant [33]. MSHQ bother decreased by 0.3 and 0.7 points at 3 and 12 months, with the decrease at 12 months reaching statistical significance (p < 0.01). IIEF-15 scores remained unchanged. In a study evaluating men aged 50–70 with catheter-dependent BPH, it was found that there was no statistically significant change in IIEF-5 scores at 3- and 6-month follow-ups [42]. An additional study with similar inclusion criteria to WATER found no significant changes in MSHQ-EjD or IIEF-5 scores at 1, 3, 6, and 12 months post-treatment [39]. One study in the Japanese population did observe statistically significant changes in MSHQ-EjD and IIEF-5 at 3- and 6-month follow-ups; however, they reported that meaningful interpretation was limited due to a small sample size likely related to a low percentage of sexually active participants at 22.3% [43].

One study compared RWJA to HoLEP and found that MSHQ-EjD scores decreased less in the RWJA group at 3 months (p = 0.02) [36]. Although differences were observed at 6 and 12 months, statistical significance was not reported.

### Preservation of antegrade ejaculation

The assessment of antegrade ejaculation preservation demonstrated the most heterogeneity in evaluation methods. For example, in the earliest trials by Gilling et al., preservation rates were reported either based on adverse events or the IIEF-15, with neither trial noting any RE [6, 40]. In the WATER trial, at 6 months, antegrade ejaculatory preservation was 90% for RWJA vs. 64% for TURP (p < 0.01) [26]. Prostates 50–80 mL showed even a greater disparity in preservation rates (98% for RWJA vs. 59% for TURP, p < 0.01) [26]. These rates improved when post-treatment cautery was avoided (93 vs. 84%, p = 0.26) [26]. For men with larger prostates in the WATER II trial, antegrade ejaculation was preserved in 81% of patients [31]. Elderly men experienced similar rates of EjD when compared to younger men (9.7 vs 12%, p > 0.05) [30]. In both WATER and WATER II, antegrade ejaculation preservation was reported as a function of the MSHQ-EjD.

FRANCAIS WATER and OPEN WATER also reported antegrade ejaculation as a function of the MSHQ-EjD, finding rates of 73.3 and 92% at 12 months, respectively [32, 33]. Two studies evaluated ejaculatory function using the MSHQ-EjD but only reported the preservation rates: one observed 73% at 3 months in a cohort of 118 patients, while the other reported 82.8% at 3 months and 84.6% at 12 months in 59 patients [37, 38]. Another study utilizing the MSHQ-EjD reported a preservation rate of 97% at 12 months among 109 patients [39]. In the study comparing HoLEP to RWJA, the MSHQ-EjD was used; however, the specific rate of antegrade ejaculatory preservation was not reported [36]. Finally, the study in the Japanese population used the MSHQ-EjD without reporting the results but noted no new cases of EjD based on patient-reported adverse events [43].

The remaining studies relied on adverse event reporting: one reported a 75% rate at 3 months, one retrospective study found a rate of 72% at 3 months among 53 patients, another retrospective study reported 81.4% at 12 months among 175 patients, and one of the largest cohorts (n = 330) with the longest follow-up (48 months) reported a rate of 99.6% [41, 34, 35, 42].

## Discussion

In this systematic review, we provide a comprehensive evaluation of sexual health outcomes following RWJA, with a particular focus on EjD and the preservation of antegrade ejaculation. As a true surgical procedure, RWJA uniquely occupies a middle ground between traditional surgeries like TURP, which have high rates of EjD, and MIST procedures like PUL and WVTT, which are office-based and guideline-recognized for their sexual side effect profiles [7–9]. The preservation of ejaculatory function with RWJA is not incidental, but rather an outcome of its carefully engineered, image-guided, and heat-free approach. The WATER trial demonstrated the RWJA procedure’s utility in preserving antegrade ejaculation compared to TURP [12]. Among the ten other included studies that reported antegrade ejaculatory preservation rates, none documented a rate lower than 72% [31–35, 37–42]. This favorable outcome represents a significant improvement compared to reported rates as low as 33.9% with TURP [4]. The WATER II trial built upon WATER and confirmed the feasibility and efficacy of RWJA in men with larger prostates [31]. The preservation of antegrade ejaculatory function was like that seen in smaller prostates (30–80 mL), demonstrating that RWJA may be a suitable option for men with large prostates who desire ejaculatory function preservation.

Longer-term follow-up data on sexual function outcomes following RWJA remain limited. Only two studies reported data beyond 12 months, one of which was the WATER trial. In these studies, antegrade ejaculatory preservation exceeded 90%, and the WATER trial showed a minimal decline in MSHQ-EjD at 5 years, suggesting the durability and maintenance of ejaculatory function over time [12, 41]. However, it is difficult to generalize these findings beyond the populations included in these studies. The WATER trial, being the only RCT on RWJA to date, has the least risk of bias of the included studies. However, out of the 116 patients in the RWJA cohort, only 58 remained enrolled at 5-year follow-up. Therefore, conclusions regarding the durability of ejaculatory function in those who did not reach this time point cannot be determined. Furthermore, the study completed by Omidele et al. [41], which followed 330 patients up to 48 months (250 reached the 48-month visit), did not report the number of sexually active individuals at baseline and determined antegrade ejaculatory preservation through self-reported adverse events [41]. This contrasts with the WATER trial, in which EjD was determined through assessment of the MSHQ-EjD, making it difficult to directly compare the two studies. Although current evidence suggests that ejaculatory function remains preserved in the long term, it remains possible that the preservation of distal apical tissue, which is critical for antegrade ejaculation, could lead to a trade-off in urinary symptom relief over time. Further prospective studies with extended follow-up periods and consistent reporting measures are needed to confirm both the durability of ejaculatory function and the long-term efficacy of RWJA in relieving LUTS.

Only one study included in this review compared RWJA to other treatment modalities besides TURP [36]. Compared to HoLEP, RWJA showed better preservation of ejaculatory function, particularly at short-term follow-up at 3 months. While this study had a small sample size, it is consistent with prior studies evaluating EjD after HoLEP. Previous studies have demonstrated RE rates of 70% or higher, a significant increase compared to findings in the present review, suggesting RWJA’s superior efficacy in this regard [46, 47]. However, despite a major aim of RWJA being the preservation of ejaculatory function, WVTT [9] and PUL [8] remain the only treatments specifically recommended by the AUA for men prioritizing the preservation of erectile and ejaculatory function (Conditional Recommendation; Evidence Level: Grade C) [7].

A significant limitation of this review was heterogeneity among the included studies. Not all studies utilized the MSHQ-EjD to assess EjD, with some reporting only the absence of RE as a patient-reported adverse event categorized using the Clavien-Dindo classification. One study reported EjD as a function of the IIEF-15. This limits direct comparisons across studies and has the potential to introduce bias into the analysis of results. Several studies lacked baseline data on sexually active participants, complicating the assessment of functional changes after RWJA using the MSHQ-EjD and IIEF-15. Additionally, most studies reported only the entire score of these surveys without drilling down on specific subcategories. If they were separated, it was often in graphical form, preventing extraction of the specific scoring. Only two studies included MSHQ bother results, which can help differentiate symptom severity from actual patient impact. While EjD can be distressing to some patients, it is not life-threatening and may be inconsequential to those who develop it. The bother score provides a useful metric to evaluate this. Future studies should ensure standardized reporting of validated sexual function metrics, with the inclusion of each category and baseline sexual activity status to improve the validity of findings. These questionnaires are not without their limitations, as they are subjective and based on patient answers and understanding of the questions posed to them.

## Conclusion

This systematic review provides an important update on the literature regarding RWJA and sexual health outcomes. To our knowledge, this is the first review to incorporate long-term data beyond 12 months. Since the adoption and inclusion of RWJA in current AUA guidelines, additional studies have reinforced its low rates of RE compared to TURP, regardless of prostate size. Additionally, current studies suggest that the efficacy of RWJA in preserving ejaculatory function may be superior to that of other therapies, such as HoLEP, with greater long-term efficacy. However, while RWJA has shown promising results in the preservation of ejaculatory function, current AUA guidelines do not recommend this therapy in those looking to preserve erectile and ejaculatory function. Therefore, further research is needed, particularly RCT studies that have a focus on EjD. Ultimately, RWJA has demonstrated promising results, and this systematic review strongly suggests it to be an effective long-term treatment option for men seeking to preserve ejaculatory function, regardless of prostate size.

## Data Availability

All data included in this manuscript can be found within the published article and through accessing the references.
